# “Vital”: HIV counselling and testing staff’s views of addressing mental health with HIV in Uganda

**DOI:** 10.1186/s12913-020-05881-4

**Published:** 2020-11-10

**Authors:** Faith Martin, Winfred Nalukenge, Oucul Lazarus, Josephine Birungi, Janet Seeley

**Affiliations:** 1 Department for Health, University of Bath, Bath, BA2 7AY UK; 2 MRC/UVRI and LSHTM Uganda Research Unit, P.O.Box 49, Entebbe, Uganda; 3 The AIDS Support Organisation, Mulago Hospital Complex, P.O BOX 10443, Kampala, Uganda; 4 London School of Hygiene and Tropical Medicine, London, UK

**Keywords:** HIV, Qualitative, Uganda, Counselling, Mental health

## Abstract

**Background:**

Mental health is linked to HIV outcomes, including linkage into care and adherence to medication. Integrated care for mental and physical health is recommended. HIV testing and counselling sessions represent an opportunity to implement interventions to address mental health, however it is first necessary to understand the roles, current practice, knowledge and attitudes of the testing and counselling staff.

**Methods:**

This qualitative study used semi-structured interviews with HIV testing and counselling staff at four centres of a HIV healthcare provider charity in Uganda. Interviews focused on their current practice, perceptions of mental health and their role in supporting this, challenges of this work, training and support needs, and views of potential greater emphasis on mental health work in their role. Data were audio-recorded, transcribed verbatim, and analysed thematically.

**Results:**

Data from twenty-one testing and counselling staff revealed five themes. Clients presented to counselling staff with needs spanning bio-psycho-social domains, where psychological health was intertwined with HIV management, medication adherence, and seen as “inseparable” from HIV itself. Mental health was largely thought about as “madness”, identifiable from extreme behaviour. As such, common mental health problems of anxiety and depression were not often seen as part of mental health. Approaches to intervening with mental health were seen as behavioural, with some ideas about changing thinking styles. Participants demonstrated significant practice of common techniques to address mental health. Needs were identified for further training in suicide risk assessment and identification of depression, together with greater clinical supervision. Participants described significant conflict within their roles, particularly balancing time demands and need to achieve testing targets against the need to offer adequate mental health support to clients in need.

**Conclusions:**

HIV testing and counselling staff described a diverse role that already includes addressing mental health. Mental health is “vital” to their work, however the time needed to address it is at odds with current testing targets. They require more training and resources to effectively address mental health, which is vital to optimising HIV outcomes. Interventions to integrate mental health support into HIV testing and counselling sessions need to be further researched and optimised.

## Background

Mental health problems are common among people living with HIV, including depression, anxiety disorders, mixed-depression and anxiety, and suicidality [1–5]. Anxiety may relate to social phobia or be at distressing levels but not meeting a specific diagnostic category [2]. This is particularly important as depression and anxiety are associated with poor linkage into care, poor adherence and retention in HIV care, greater risk of transmission, and poor outcomes for the individual [5–9].

Mental health is linked to the achievement of the UNAIDS’ “90–90-90″ HIV targets to end the epidemic by 2030. These targets are to achieve diagnosis rates of 90%, with 90% of those diagnosed on anti-retroviral therapy (ART), and 90% of those people with an undetected HIV viral load by 2020 [10]. Huge efforts have gone into increasing access to and uptake of HIV tests to address the targets, including calls to “integrate” mental health care into testing, to support linkage into care [5, 11–13]. Despite its relevance to joining and staying in care, addressing mental health is not a major part of international HIV policy. Ugandan guidelines have more recently suggested routine screening for depression [14], however the extent to which this is part of routine practice is unknown.

Uganda had a 5.9% adult prevalence of HIV, and data suggest that 89% are on treatment, and 78% are virally supressed, based on data collected in 2017 [15]. However, there is significant variation between different segments of the population. For example, a study with female sex workers found of the 31.4% of the sample who were HIV positive, just 45.5% knew their status, 37.8% self-reported they were on HIV treatment, and 35.2% were virally suppressed [16]. There are issues in relation to linkage into care, with studies reporting suboptimal rates of people starting anti-retroviral medications and staying in HIV care. One study in Uganda found just 53% of patients linked into care (i.e. registered with a facility providing treatment) within 1 month of their HIV counselling and testing (HCT) session [17]. Using home-based testing with augmented counselling in Uganda, another study achieved 44% linkage into care [18].

Time taken to link into care may reflect pathways from community testing to engagement with clinic services, and challenges in service delivery and accessibility [19]. Further, linkage into care may simply take time: people need to accept and adjust to the diagnosis, reconcile how to manage stigma and make practical arrangements [20]. This may be lengthened by unmet mental health needs, amongst other potential drivers including health system, community, financial, household and individual factors [17, 20, 21]. Mental health support may facilitate linkage by treating anxiety and depression, and addressing acceptance and stigma issues. “Test and treat” or “test and start” policies, which advocate immediate treatment [22], are not readily compatible with this need for longer-term processes of treatment and acceptance [23]. People need time to adjust to their diagnosis and their identity as someone needing to take lifelong, daily medication.

The first step of linking people into care, and maintaining engagement once linked, is the person’s first step into the HIV system: their initial HIV counselling and testing appointment (HCT). The aims of HCT include case detection, reduction of post-test risk of transmission and increase linkage into care [24]. HCT also presents an opportunity to address mental health, for its own sake; as part of the movement to increase access globally to mental health care [13]; and to bring the person into the cascade of care [25]. The HCT contact is a crucial moment to give people this preparation and support they need to then be ready to embark on a lifetime of treatment [26]. This may involve screening and addressing people’s mental health [27].

The World Health Organisation (WHO) 2015 guidance emphasises the “5 C’s” of counselling, being “consent, confidentiality, counselling, correct results and connection [into HIV care]” [24], extended by some to include “consent process” needing to be convincing and “couple counselling” as beneficial [28]. Some researchers highlight that the focus has been on the technical aspects of testing, rather than the complexities around offering appropriate support [29] or taking into account the social implications of a HIV diagnosis that can impact linkage to care [30].

There is a tension between movements towards more rapid testing and self-testing approaches, versus the recognition of the need for mental health care. On one hand, there is a push to increase numbers tested using abbreviated, home-based and self-testing approaches. These approaches do not address mental health and may have lower rates of linkage into care [8, 31–33], however are attractive in that they appear initially to progress towards 90–90-90 targets. Furthermore, some areas of sub-Saharan Africa have experienced reduction in provision of HCT, despite the remaining need [34]. There is a pressure for rapid and low resource approaches to counselling and testing. Indeed, WHO 2019 guidelines for HCT suggest concise behaviour change messages as the focus for post-test counselling [35]. On the other hand, there is recognition of the need to include greater mental health care, integrated into testing sessions, [36, 37]. This may require more counselling time per person, reducing numbers that can be tested per day. On the surface, in the short-term, this may appear to limit progress towards 90–90-90 targets, however time spent on counselling may increase linkage and retention in care in the longer term and thus be worth the investment.

The views of current HCT counsellors are instructive to our understanding of if and how to develop their role to include more mental health support, and the extent to which they see this as a “development” or existing practice. This study aimed to explore HCT counsellors’ views of their role, attitudes and knowledge around mental health, and perceptions of the impact and requirements of extending their role.

## Methods

### Setting and participants

The sample for this study was drawn from counselling staff working for “The AIDS Support Organisation” (TASO) in Uganda. TASO is one of the nation’s largest indigenous non-government organisation, providing HIV counselling and testing, care, treatment and psychosocial support to HIV positive persons and their families. Around 80,000 HIV positive adults received direct input (testing, treatment and support) each year (TASO, personal communication based on internal data).

We recruited participants from four centres, to provide some diversity and remain accessible to the researchers. Each centre has around 12 counsellors. Recruitment was designed to provide a range of views. We planned to recruit 1:3 male: female, to mirror staffing ratios and to recruit 4–8 staff from each site. Counsellors currently employed to provide HCT to adults were eligible to participate. We excluded 1) “expert clients” who are engaged in providing basic counselling/information 2) part time counsellors and 3) interns on placement to gain counselling experience.

### Data collection

The lead author visited the research sites and introduced the study to the teams. Voluntary participation was emphasised. Subsequently, participants were invited to volunteer to complete a semi-structured interview with a Ugandan research assistant. The research assistants all had training and experience conducting interviews of this type. They all met with the site managers and spent time at the research sites to conduct the interviews and complete the wider data collection for other components of the project this study is part of (specifically, questionnaires with counsellors relating to skills used in HCT sessions and observations of HCT sessions). Informed consent was gained from participants. An interview schedule was used to guide the discussion and provide prompts. This contained a prompt to explore views of their current practice, role in supporting mental health; understandings of mental health; challenges faced in their current work, particularly relating to their broader socio-cultural context (e.g. their own identity within their community); training and support needs; and perceived challenges and opportunities in upscaling their delivery of mental health interventions. The questions are based on the research aims and previous research exploring the experiences of HCT staff [38–41]. Interviews were conducted individually, and in English, as all were fluent in that language. The initial interview schedule was altered after it rapidly became clear that it was necessary to prompt participants further to consider “psychosocial” issues within their consideration of “mental health” and to describe the concept of clinical supervision (more details provided in the results section).

### Data analysis

All interviews were audio-recorded and transcribed. Data were anonymised, with participant codes used and removal of any details that would identify any of the clients the participants spoke about. The lead author completed the analysis. The lead researcher is a British psychologist, with clinical experience working with people living with HIV and HIV care teams in the UK, and previous research experience in relation to HIV and psychological wellbeing in Uganda. An essential step, owing to the cultural differences between the lead and the research participants, was to discuss analysis with the second author (both checking minor details and sharing and critiquing broader themes), who led the data collection, and with the wider research team and authors. Interview data were subjected to thematic analysis [42]. Data analysis was managed by hand, with a combination of notations using the “comments” function in Microsoft word, copying of sections with shared codes into Excel and handwritten notes to collate the codes and generate themes. Transcripts were initially read and re-read. Initial thoughts were noted at this stage covering any initial impressions and queries about the data to be clarified with the second author. Next, transcripts were open-coded, without reference to any particular theory, although of course influenced by the researcher’s background as a clinical psychologist. These initial codes were collated and themes created. For example, initial codes relating to concepts around the job role and challenges of time, resources and so on were joined to form the theme “conflicts in role”. Themes were reviewed and at this stage data examined again to explore any “deviant” examples, such as identification of a spectrum of difficulties by some participants in contrast to the more dominant view of mental health relating to only extreme problems. Training and supervision needs were combined into a single theme at this stage, as they reflect linked issues regarding staff development. The final themes were then defined and shared with co-authors.

### Ethical considerations

Ethical clearance for this study was granted by the Uganda Virus Research Institute and the University of Bath. Informed consent was gained from all participants, who self-identified as interested in taking part. Local researchers conducted the interviews. Participants were all reminded of their right to withdraw from the study and given contact details for the research team. Participation in the study was not reported by the researchers to the participant’s managers or colleagues. Data were fully anonymised.

## Results

### Participants

Twenty one counsellors took part in the interviews, detailed in Table 1. The counsellors were typically very experienced, and this is a feature of the organisation where counselling staff are trained by TASO and continue working there.

**Table 1:** Details of participants

Location	Number of counsellors	Number female	Mean years in counselling role
Entebbe	4	3	6.8
Jinja	6	3	9.0
Mbarara	5	3	9.4
Mulago	6	2	8.3
TOTAL	21	11	8.5

### Themes

Five broad themes were identified from the data: Diverse and complex client needs; “madness” as behaviour; approaches to “change mentality”; training and supervision needs; and conflicts within role. In the presentation of quotes below, no identifying information is provided in order to preserve the anonymity of respondents. Themes are presented with sub-themes. We integrate our discussion of results below.

#### Diverse and complex client needs

##### Range of needs

Participants named a wide range of clients’ needs, often given as lists of the issues they work with or using brief examples. Biological/medical issues such as testing, other infections, and problems with medication were common. Psychological factors included low mood, fear, anxiety, and suicidality. Family, social and work needs were common including housing, conflict, legal issues and brushes with the police, access to land, and social issues of living in environments of poverty and violence. Describing only health related needs, one participant noted the many dimensions of this one area:

“Most of the time when the client comes here we talk about drugs, we talk about nutrition, we talk about appointment, why didn’t you come for your treatment , why didn’t you take your drugs in time , why are you losing weight. T. B prevention, such things you know. We talk about TB prevention, nutrition, and those medical things then work, weight, height, what else aaaah doing viral load, doing CD4 tests.” (Man, Jinja).

One female participant from Entebbe described how clients come to the counsellors with multiple needs, including housing concerns, new business plans they would like support with, financial difficulties, and access to food. Her advice ranged from exploring advantages and disadvantages of different businesses and suggestions to “grow greens in that [their land] space [rather than] struggling going to market there they cannot afford to buy things”.

HIV and ART (referred to by some participants as “ARVs”) was ‘just’ one amongst many concerns for many clients:

“This is an old lady, she has no land so there is a question of food insecurity, she is on ARVs, social support zero because even like the relatives are also poor, needy they cannot support her” (Man, Mbarara, as above)

Again, illustrating the complex needs and challenges in life, one participant told the story of a young woman with a baby that they had worked with for sometime.

“The baby had taken two days without eating food and the [young woman’s] mother was violent and yet the grandmother also had no food and no basic necessities for the girl to use so she was requesting for money to buy for the child milk and buy a saucepan and other things to use in the hospital, so I had to contact the organization that had to take over the baby so that it gets to be fed.” (Man, Jinja)

##### Chains of needs

People talked about chains of needs where stress may trigger other difficulties:

“Because when you are drunk you are not going to take your ARVs in time and ah … that also triggers gender-based violence”. (Man, Mbarara)

Ill-health, owing to HIV, can lead to loss of employment and increasing poverty “The person might be very sick and cannot even do casual labour to earn some income because those that are not very sick can work for people and earn some money” (Woman, Entebbe).

In their conversation, participants linked the meaning of HIV to other difficulties. A HIV diagnosis, suspected by others or confirmed, was seen as making a person less attractive as a spouse, and therefore there were implications for social status, and reduced likelihood of having a ‘normal’ family owing to stigma. The social meaning of HIV continues to carry risk, and the worry of this risk and being found out could be linked to stress and mental health. Providing an example, a participant stated

“A client comes and tells you my husband has thrown me out of the house, I don’t have where to go, all my parents died and I don’t have any relatives meaning that person is going to go on the streets that’s one situation, another person comes and tells you when I told my officer at work that am HIV positive they are now threatening to chase me away so that’s also another problem”. (Female, Entebbe)

##### HIV and mental health needs

Explicit links were made between HIV, ART and mental health: “Adherence is another problem because when they are mentally disturbed adherence is poor”. (Woman, Entebbe).

Participants described a strong link between HIV and mental health, identifying how crucial this was to retention in care, adherence to medication and survival. When asked how mental health fits within the HCT role, one participant stated it is “Vital in our day to day activities” (Woman, Jinja). Another participant described why this was:

“Mental health, even like psycho-socio sciences they are almost like inseparable … we’ve seen even some of the death in HIV caused by mental health issues because yes they are ARVs but somebody is going to fail with HIV, he drops out of care he or she dies” (Man, Mbarara)

The experience of the counsellors, and their reflections of their clients’ experiences, is that HIV, mental health, and wider life concerns are intertwined. Their role is complex, addressing multiple client needs, including attempting to address mental health. The “test and treat” policies relate solely to testing for HIV and offering ART to treat HIV. What appears to be needed is a “test-understand-treat” policy: test for HIV, understand the person’s situation, and offer treatment for HIV and for wider psychosocial issues.

#### “Madness” as behaviour

A number of participants reflected on the ways in which language was used. One participant noted “Mental health are English words, but us, we have what we call psychosocial” (Woman, Mbarara). During the interviews, some participants reflected that they had not “realised” until the interview that they were working with “mental health” issues, having previously seen this as something exclusively related to more extreme “madness” requiring referrals to the specialist psychiatric hospitals. Ideas about mental health are culturally mediated [43]. As a British Clinical Psychologist, the lead author had conceptualised “mental health” as meaning psychological health, including the distress, despondency and sadness that may normally be experienced when managing chronic illness [44], as well as more extreme emotional difficulties that may be given a psychiatric diagnosis. It is important to recognise the continuum of distress to psychological problems that impact functioning [45], which may require a continuum of support and intervention.

Mental health was talked about in a variety of ways. The overall theme highlights the finding of a focus throughout on behaviour. The subthemes cover the different ways mental health problems are understood, detected and considered as a discrete set of issues or as a continuum, in addition to how mental health is linked to HIV.

##### Understandings of mental health

There was a wide range of attitudes to and knowledge of mental health. There was mostly a lack of “western-based” or medical model-based knowledge about mental health. Participants described ideas of severe distress and widely held community views of mental health as linked to witchcraft, financial problems, or HIV itself.

“They [people in the community] assume it [the mental health problem] is a family background; they associate it with financial background. It is associated with witchcraft; sometimes it’s attributed to HIV.” (Woman, Mulago)

Here is a clear example of how the counsellors situate distress in the individual’s context. This viewpoint is sometimes cited as unhelpfully missing in dominant psychiatry/psychology approaches in the UK and USA [46].

##### Detecting “madness”

Mental ill-health or difficulties were often linked to “madness”, a term frequently used by participants. They spoke of people “with incoherent thoughts about her life”, who may be “running mad”, which might be identified by “having uncoordinated statements, making noise”. Participants meeting people with extreme problems including suicidality and what appeared to be psychotic illnesses.

“I have had a scenario where one was HIV positive and they had a daughter so what they did is prick [stabbed] herself and also pricked [stabbed] the daughter too because they were worried about who would take care of the daughter if they died.” (Woman, Entebbe)

Identification of mental health difficulties was then inferred almost exclusively from observable, unusual, “funny”, violent, or excessively talkative behaviour. In many cases mental health difficulties were identified on the basis of transgression of socially normal behaviour. At times this was personalised: odd behaviour for that individual, highlighting the importance of counsellors having time to build relationships with their clients to be able to “read” them.

“It depends on someone’s behaviour like some clients we know about their behaviours so if we see them behaving in a funny way or talking in a funny way we can say this person might be having mental health difficulty.” (Woman, Jinja)

Participants based their views primarily on the most extreme cases, with “funny” (unusual) behaviour being the indicator of mental illness. Emotions or thought patterns were not described as indicators of mental health/illness, suggesting a lack of recognition of the roles of cognition in distress, and a lack of focus on the actual experience of distress. This is similar to previous research, finding that in general communities are concerned by behavioural disturbances, which they name in some way as madness [47].

##### Continuum of mental health

Different levels of severity of mental health difficulties were discussed by some counsellors, but this appreciation of a spectrum of difficulties was named by a minority of those interviewed.

“There are those clients who have mental health difficulty at a low level but there are those who have it at a higher level whereby even if he or she does not speak the character will speak for itself.” (Woman, Entebbe)

“It is a big spectrum; I’m not looking at psychosis and alike. It is anything that challenges my mental facilities: stress, depression, anxiety and euphoria.” (Man, Mulago)

The identification of distress at different levels suggests there is potential for different types of mental health support, and counsellors may already be offering a significant amount of psychological support and intervention at the “low level” of distress. The meaning of “low level” is somewhat ambiguous. “Common mental disorders” include major depressive disorder [48], which has a prevalence of around 8% in Uganda in people with HIV [3], and there will be more scoring with milder forms of depression, not to mention anxiety disorders. It may be that these peoples’ distress is not being identified nor addressed.

##### Links between mental health and HIV

Strong links were described between HIV and mental health. One woman in Entebbe stated “Mental health can result into HIV and HIV can result into mental health so it is a correlation of the two so it can fit well into our setting”. She illustrates bi-directional relationship between HIV and mental health. The impact on mental health of living with the stress of HIV was described by a man in Mulago: “We are aware that they [people with HIV diagnosis] tend to lose hope, support and friends and develop negative emotions.” Fear of the unknown, denial, stigma and non-disclosure were listed as direct impacts of HIV on mental health by one man in Entebbe.

A very direct link was described between HIV and mental by one woman in Jinja for example. She stated that “HIV may go on their brains” causing mental health problems. This demonstrates HIV as a perceived biological cause of mental health problems. The participant did however also acknowledge that the cause may be due to “the effects of being worried, feeling sad” because of their HIV diagnosis. There is an existing body of work on explanatory models of mental health issues (for example, [49–51]). Here, the data showcase the way participants see mental health and HIV as firmly linked issues.

Mental health difficulties were linked HIV medication. Very occasionally, participants spoke of ART as causing mental health problems, however this was rare and typically relate to the use of Efavirenz. For example, a woman in Entebbe told the story of a client who adheres well to their ART, but as has been reported with Efavirenz [52], they developed memory problems and confusion, leading to an incident where the client was naked in public, and they was taken to the mental health hospital and following a change of ART, they recovered.

More frequently, participants spoke about the link between mental health and lack of adherence to HIV medication, often talking about this very briefly in fragments – for example one woman in Mbarara named the example about people forgetting to take their drugs due to alcohol misuse and also mentioned, when naming clients’ needs and topics of discussion, “psycho-social” problems that “can make someone to fail to take his drugs”. A man in Mulago described how patients with mental health problems forget to take their ART. He also provided an example about a female client who did not take her ART as she had mental health problems. He described her mental health problems as characterised by behaving aggressively to others and “making weird demands”, for example “instead of taking warm food she wanted ice food”. He linked this woman’s mental health problems to being unable to take her medication, however he did not elaborate on this link. It is not clear from the data the exact mechanisms by which participants think mental health reduces adherence, with “forgetting” being the common explanation.

Participants made the fundamental link between mental state and HIV clear, re-emphasising the importance of mental health to the HCT role.

#### Approaches to “change mentality”

##### Challenges to intervening

Counsellors described mental health interventions as being time-consuming. A focus on creating changes in “thinking” and “mentality” was observed, with a requirement that interventions are individualised. Typically, participants felt lacking in skills to deliver interventions, but confident in referring clients with “mental illness” (here obvious suicidality and ‘psychosis’) to psychiatric services. Difficulty was seen in addressing complex mental health needs: “If a patient is totally mad, you can’t apply your training”.

The lack of mental health awareness in the community and beliefs that mental health difficulties are caused by “witchcraft” were challenges to creating change. One participant succinctly put it

“So if we are to address the issues of mental health we must first of all address the myths around mental health.” (Woman, Entebbe)

##### Interventions for mental health

Techniques and approaches used included offering active listening, empathy, encouragement, curiosity, and an opportunity to express emotions. Interventions were sometimes cognitive in nature: “We encourage them to focus on changing their mentality”. Most participants talked about generating a sense of hope or positivity. Specific cognitive techniques were described by some, for example:

“Here is a technique of externalizing where you separate the problem from the person, yes we can have problems but we need to move on because when you make a problem part of you then you can never move on.” (Woman, Entebbe)

These core or common factors form the bedrock of most forms of psychological therapy and are significant active ingredients in interventions aiming to reduce distress [53]. These techniques are common across a range of therapeutic approaches, and are important to address mental health difficulties in terms of normal distress and psychiatric diagnosis, and basic competences for those working in mental health [54]. As such, the presence of common factors in participants’ reports of their work suggests mental health intervention is already part of their work, be it not necessarily a formally acknowledged element.

Much of the work was behavioural, in terms of encouraging staying busy, being social and continuing to set goals in life. This could be instrumental to improving their situation, or to tackle rumination.

“So if clients have issues stressing I tell, them sit down, have plans, think of investing and doing other things other than thinking about that problem every single day” (Woman, Entebbe)

For some, this was linked to “empowering” clients. Many participants talked about the importance of family, often centring on alcohol misuse. Interventions focused on behavioural changes in the family to ensure help was provided, and helping people access community resources where there were financial challenges. A range of therapeutic approaches are in evidence: behavioural therapy, some basic cognitive therapies and family/ systemic approaches. There exists a foundation of intervention knowledge to be built upon with further training to provide a range of options to support clients. A person-centred approach to intervention is evident, with a desire to empower clients to look after their HIV and mental health.

##### Specialist services

Generally, participants viewed mental health as a component of good HIV counselling. However, a different view was seen where participants considered more severe mental ill-health, seeing a need for more medical treatment and formalised care. In contrast to most responses, one respondent explained that rehabilitation was needed, which has strong undertones of a medical model of understanding.

“So mental health is perceived negatively but to those that have information they do not perceive it as a bad thing, it is just like HIV it affects the brain at some stage but with the support that clients receive from the counsellors and the medical support so with that whole package we believe someone can live with this virus, so with rehab mental health patients can also be supported and healed.” (Woman, Entebbe)

Another participant talked about the need for a place to

“accommodate these people … it would be a bit different from the normal one, so they will need their special place, or their own kind of site, you don’t mix them” (Man, Mbarara).

This represented the idea of mental health care as different, specialist, and removed from mainstream care. The idea of a spectrum of mental health was again evidenced, with different needs for intervention at the more extreme end, and a different role for the counsellors as referring clients on. This fits with ideas of stepped care, with general care being widely available, alongside specialist services for people with extreme difficulties [55].

#### Training and supervision needs

*Training needed:* Given the view of mental health as often quite extreme illness, there is an implicit need for training to support detection of less severe difficulties, including difficulties presenting at an early stage. Reflecting on a case to highlight training needs, one participant stated

“maybe not realizing early enough that it was a serious mental health problem that needed to be handled at another level, because there are those mental issues that go beyond what we can handle and need further referral.” (Man, Entebbe)

Staff reported frequently assessing depression and suicide risk. They reported a strong need to do this work, owing to the lack of other services that may detect these difficulties, e.g. no universal primary care service where mental health problems may be detected. Participants were not highly confident in their skills to do these assessments: “there must be ways of detecting mental health which I must be having a lay men’s way of detecting” (Woman, Mbarara). Participants talked about the need for further training in detection and assessment. The tension is seen between seeing some distress as normal, given a chronic illness and often many life challenges, versus a desire for a formal, expert approach to assessment. Again, we see the risk of medicalising distress [56], and of devaluing the human interactions between counsellor and client in favour of a clinical, “expert” protocol. The therapeutic relationship built through these interactions is essential to any further support and intervention [57, 58].

Participants varied in their reported level of confidence in addressing mental health. A common feeling was that they were supporting mental health, but not necessarily “using mental health techniques”. However, on questioning, most counsellors identified mental health related skills they were using, such as active listening and creating a positive relationship with clients. Specific treatments, such as cognitive-behavioural-therapy techniques, were not mentioned however, suggesting a strong but generic level of skill.

Format, content, duration and other details of training were not frequently discussed by participants. One suggested the training be put online so “at least we know the protocol” (Woman, Mbarara). This comment also shows the idea of mental health interventions having a protocol or manual to follow, something more formal than the current work.

##### Supervision

Counsellors reported a significant benefit from management supervision and informal clinical supervision. However, there was a perception that with greater mental health work would come a need for greater clinical supervision to ensure quality for clients, encourage use of appropriate techniques and interventions, and to support counsellor self-care. Experiences of supervision and support with client work were varied, but typically positive. Many participants reported they would like more supervision and in some cases this was arranged in an informal manner. The need for supervision was related by some to their caseload.

“We are expected to see 7 clients a day but sometimes we see as many as 10. It would be nice to share with that supervisor on your experience.” (Woman, Mulago)

The importance of supervision was clearly named by many, who referred to it as “counselling”, as the term “supervision” is not currently used in this setting.

“I believe that counsellors need counselling. ‘When you nurture a goose very well, they lay gold eggs’. When I get this type of counselling, I can get all the support I need and get back on my feet.” (Man, Mulago)

Clinical supervision was a concept we found that our researchers had to explain: initially supervision was understood as management rather that supervision to protect clients’ safety, direct intervention, and support effective therapy [59]. This version of supervision is something participants described having some access to, however wanting to be able to share their experiences more and to use this to improve their work further.

#### Conflicts within role

Two fundamental conflicts were observed: breadth of role and quantity versus quality.

##### Breadth of role

Staff described conflict in the time to spend across the tasks in their broad role, that covers medical testing, medication distribution, health behaviour change work, and addressing psychosocial issues. Most participants were clear that the psychosocial /mental health support they provide covered both psychological wellbeing and behaviour change as two, fundamentally interconnected goals. Participants talked in the same breath about health behaviours and mental health, for example ““Mostly I can talk about adherence then the psychosocial surrounding” (Man, Entebbe), and “So when there is a new client enrolled, is just coming to get refills possibly he has just come for only ARVs or has some issues which are psychosocial” (Man, Mbarara). One participant summarised this as “we swing between the medical and psychosocial departments” and the impact of time spent with clients.

“Sometimes we are torn between medical things for the good of the client or counselling and in the end we have not given our clients enough time for counselling because we have added on new things but the capacity doesn’t increase”. (Man, Jinja)

The participants recognised the need for attention to multiple issues to support HIV related outcomes and client’s wellbeing. There are competing, interlinked priorities counsellors’ felt were part of their role, and only limited time to complete their work.

##### Quantity versus quality

Driven to see more and more clients, to contribute to meeting the 90–90-90 targets, counsellors had larger caseloads to manage, reducing the time they had available for each individual. Counsellors talked of the need to give clients time to be able to attend to mental health relating to the shock of diagnosis, experiences of stigma, or challenges of life-long medication adherence. This was in conflict with policies from funders to process large numbers of clients, particularly because mental health intervention was seen as time consuming: “Patients require a lot of patience; you are able to then see some of those things come out” (Man, Mulago).

The concern that this in effect lessened the impact of their work was named by some: offering more clients less intervention was seen as potentially ineffective. Many participants talked about this, emphasising the impact on quality, which is some cases was being felt by the clients. The targets themselves were outlined by one participant.

“We work to meet targets and the number of clients is increasing for example you can register like 10 new HIV positive clients in the system, these people need a lot of time because they are new so you need to counsel them because they all have different issue” (Woman, Entebbe)

The resulting lack of time for people was described by one counsellor:

“Of course, when they are many [people to see] you can’t give wholesome quality service. You would provide wholesome service but you would need to get time and compromise. So if they are many you don’t concentrate on quality. You can concentrate on giving group sessions but of course with group sessions there are things you will leave out. So you would compromise on quality” (Man, Mbarara).

Here, the participant reflected on the need to balance the conflict between quality and quantity with a compromise, however this compromise appears to lead to reduced quality. Indeed, one participant reflected that reduced quality was noticed by clients. This participant highlights the small number of people he perceives as receiving counselling.

“People complain that these days counsellors don’t have time for clients and also about the quality of counselling but all that went when they combined these two roles [medical and psychosocial] so I don’t know but I wish that we would go back to that. I gave you a scenario of 150 people and you find only 40 counselled which is not 50% of the clients who have come on that particular day.” (Man, Jinja)

The lack of time for counselling was seen as particularly challenging as mental health support is seen as timeconsuming by one man in Mulago. He went on to reflect that this means “I may not really be there for these people” (Man, Mulago).”.

When talking of targets, participants commonly focused on quality of service and the personalised nature of their talk about clients faded. They focused on system processes, for example giving a service, registering, and completing counselling at a specific rate. These processes risk taking over from the attention paid to the individual and their needs, and the building of essential relationships with clients. The power targets hold is clear: even job descriptions are trumped by targets. The emphasis was again on time: a lack of time to give adequate service as more people needed to be seen.

It is important to note there was no alternative discourse about the impact of targets evident in the data. There was a potential solution offered, however: a male counsellor from Mbarara made a suggestion regarding service pathways: enrol the person in care, then offer mental health intervention as a special, separate programme.

## Discussion

Our findings from this study revealed five themes. First, counsellors talked about the diverse and complex needs that clients presented with. Issues included medical problems, stress, family conflict, unemployment, stigma, alcohol/substance use, and financial problems. These experiences mirror previous research in this area [40]. Mental health was seen as “inseparable” from the clients’ needs, and from their HIV management. Therefore, HIV counselling was inherently linked to mental health work. It is poorly fitting then to suggest that HCT should not include mental health. Second, identification of mental health was complex and must respect cultural idioms. Third, counsellors described a range of approaches to improving mental health already, often including fundamental “common factors”, which underpin most psychological interventions. This range could be broadened, linking to our fourth theme around training. Training could further counsellors’ skills in psychoeducation for the public, and more specific, evidence-based techniques, as well as ways to identify distress. Finally, counsellors experienced conflict in their role, relating to clients’ competing needs and the tension between quality and quantity of sessions delivered.

The complexities of terminology and conceptualisations of psychological health and ill-health were uncovered. We used the term “mental health”, intending to be neutral in valence, neither well- or ill-being. Participants explained they use the term psychosocial instead. When we then asked about knowledge of mental health, participants thought about mental illness, in its extreme forms. Some participants talked about how they had not identified distress, unhappiness, and stress as “mental health” and therefore did not link their knowledge and work in this area was indeed mental health. Participants also talked about a need for interventions to educate people about mental health, to “address the myths around mental health”. Previous research with primary care practitioners doing HCT in Uganda reported a lack of mental health knowledge [60]. What is meant by “knowledge” here is important: to ensure cultural relevance it will be vital that education interventions to dispel myths must first understand local mental health conceptualisations and provide culturally sensitive information, that takes social conditions and beliefs into account. Participants saw their work as supporting people’s psychosocial realities, rather than efforts to ‘treat mental health’. The former contextualisation of distress must remain an important part of future mental health support that may seek to also “treat”.

The theme of conflict had implications for HIV care systems. There was a tension in performing their role, centred on the push to see as many clients as possible versus the desire to offer high quality intervention that also addresses mental health. There is a lack of research into the impact of shorter forms of testing and counselling on mental health, and the extent to which that then impacts linkage into care and viral suppression. Some suggest that taking time to consider and screen for depression, for example, may improve linkage into care [9]. Our participants explained, from their experience as workers, how vital mental health is to HIV outcomes. They talked of how the quality of their work to address mental health is at risk, suggesting a potential knock on effect on HIV outcomes. For counsellors, mental health and HIV are *integrated* in that clients’ present their intertwined life struggles, and counsellors seek to support these. It can be argued that service design has lead to *disintegrated* mental and physical wellbeing services. It is now essential to *reintegrate* mental and physical health by designing services that do not artificially segment people’s lives.

The 90–90-90 targets are due in 2020, and appear unlikely to be met across the board in Uganda, with low rates of detection, treatment and suppression in particular high risk populations [16]. Thereis an imperative then to optimise testing and counselling protocols, which currently offer no specific guidance in relation to mental health. Despite a clear statement in guidelines that quality assurance of both testing and counselling should be made a priority [61], it is also noted that whilst “HIV test can be easily described through a series of steps and instructions (procedures, protocols), counselling services are more difficult to standardize” [62] . Current HCT guidelines simply suggest screening for mental health problems and referral on [63]. Recent guidelines on HCT state it should focus on motivational messages in post-test counselling [24]. The 2019 WHO guidelines both push for concise communications at post-test counselling with emphasis on linkage to care, and state that “people who learn their status without adequate support may not link to care” [35] (p3). This somewhat mixed message echoes the perceived role conflicts described by our participants.

More work is needed to specify how to best support mental health difficulties, with limited introduction of additional steps in the cascade of care where clients be lost from services. Task shifting approaches and stepped care are suggested as solutions to improving mental health care within HIV services [13], however these rely on the person with HIV engaging with a new service or part of a service following their initial HCT appointment and diagnosis. People may get lost to service in that step to move to another service or part of a service. Brief counselling provided by the HCT staff member who gave the positive result may reduce the potential loss to both engaging in supportive counselling and then linking into care, by reducing the number of steps a client has to take to new staff and services.

A qualitative study of antenatal women in Tanzania’s experiences of HCT highlighted the lack of emotional support, concluding that “resource limitations may hinder post-test counselling from having a positive impact on health behaviour and may instead contribute to feelings of uncertainty and doubt” (no page numbers) [29]. The focus on messaging about risk rather than fuller emotional support represents a missed opportunity to support people and potentially link them into HIV care, as well as being at odds with what we find counsellors are drawn to do in their interactions with real people with real needs. Another study in Swaziland found that women reported pressure from counsellors to initiate ART, because of organisational targets, and calls for counsellors in each clinic who are expertly trained to deal with the broader social barriers to linkage into care [64]. We support and extend this to also addressing the mental health barriers.

Post-test counselling is required for all, and a homogenous approach is often described, which may have “diluted attention from effective referral to [HIV] care and treatment” (p67) [30]. Similarly, at a policy level, attention to the risks and reasons that may adversely affect a person’s engagement with HIV services has been lacking, particularly in relation to mental health. A review of HCT suggests that the role of its counselling components need to be reconceptualised, from its current focus on messaging about risk to considering the broader determinants relating to HIV risk, with quality counselling and funded post-test services [65]. Our data suggests that this is understood ‘on the ground’. We suggest therefore that the “5 Cs” of HCT [24] need a sixth - that of “conceptualisation”. An approach of test-understand-treat may be needed, to allow counsellors time to conceptualise what may be happening for the person they are working with and therefore offer, or make effective referrals to services, to support them to access care. This may include providing limited mental health services or linking people into these services. At one level this appears at worse as naïve, given a highly pressured HIV care system with limited funds, often in contexts without mental health care. However, mental health support should also be considered as an essential item, not a luxury, given the known links between HIV treatment use and mental health.

Our results do give rise to several practical, health service recommendations. First, the “vital” nature of mental health to HIV demands integration of care. It will be necessary to therefore examine the extent to which this is currently happening in routine HCT practice. Next, given the identification of extreme behaviours and high risk as being “mental health” conditions, it is important to consider how early identification and support for lower levels of distress can be developed and discussed in a culturally sensitive manner. Third, training for identification and support/treatment of mental distress is needed. Training programs should be co-designed, culturally embedded, and potentially delivered online as participants indicate challenges with time in their role. Fourth, it is essential to pay heed to counsellors comments about the unhelpful side of the targets set for them, and the perverse effects of these as potentially reducing quality of care, and potentially reducing long-term outcomes in relation to treatment initiation and retention in care. Finally, interventions for mental health in the context of HIV, that begin at the testing session must be designed and rigorously evaluated. These can build on existing research into task-shifting and stepped care models (e.g. [66]). Their evaluation should include a consideration of the cost of time needed with clients versus the potential benefit of clients having time to adjust to their condition.

This study has limitations. Although the dependability of the analysis is supported by the notes kept of the coding process and description of the methods, there are inevitably some decisions relating to the details of the themes that another researcher may approach differently. The lead researcher has experience of working with UK adults and young people with HIV and mental health problems, and carries a fundamental bias that this is an important area of work. To reduce this bias, she kept notes of her initial impressions to highlight biases and “deviant” cases were sought where alternative ideas were intentionally sought. Further, checking with the lead data collection research assistant and broader team redressed the biases. Transferability of findings is limited as the participants were experienced counsellors and may have different views and confidence when compared to counsellors just entering the role, or working across other organisations in Uganda where training may have less emphasis on the psycho-social. Our interview schedule used language of mental health, and the counsellors’ interpretation of this was challenging to understand. Further research could use different terms and explore explicitly views in relation to common distress and disorders such as depression and anxiety, as compared to more extreme distress that might be termed as psychosis or personality disorders. Our findings relate solely to the views of the counsellors, rather than providing quantitative support for the perceptions shared. It was not possible to enhance credibility by sharing the themes with the participants, however two of the co-authors work with TASO and have sound knowledge of the day-to-day work of the counsellors.

## Conclusions

We found that counsellors have a diverse role, where mental health is vital and critical to HIV treatment, however current targets to test large numbers of clients are creating tension for staff. Counsellors are already doing “mental health” work, as they cannot separate it from the rest of their role, therefore it must be supported by training, resource, and recognition in HCT procedures as an important step to conceptualise how a client may best be supported, and linked into care. Re-integrating mental health with HIV in our thinking about services, to match people’s experiences, is essential. Training and supervision are needed to provide care that reflects a range of levels of distress in a culturally sensitive way, without over-medicalising distress. The next steps are to develop optimised HCT that contains mental health components that are streamlined and clinically effective, and to establish the effectiveness of this on HIV treatment outcomes, distress and mental health.

## Data Availability

The datasets generated and/or analysed during the current study are not publicly available, in order to protect participant identity, but are available from the corresponding author on reasonable request.

## Abbreviations

ART: Antiretroviral therapy
HCT: HIV Counselling and Testing
HIV: Human Immunodeficiency Virus
TASO: The Aids Support Organisation
